# Supplementary material for: An introduction to DUIA: The database on urban inequality and amenities
Source: PLoS One. 2021 Jun 25;16(6):e0253824. doi: 10.1371/journal.pone.0253824 (PMC8232421; doi:10.1371/journal.pone.0253824)
Supplement: S1 Appendix — (DOCX) [file pone.0253824.s001.docx]

## **S1 Appendix. City-level databases initiatives**

| Africapolis | https://africapolis.org/home |
| --- | --- |
| Global Urban Indicators Database | <https://data.unhabitat.org/search> |
| C40 Cities | <https://www.c40.org/research/open_data> |
| Global Human Settlements | <https://ghsl.jrc.ec.europa.eu/ucdb2018Overview.php> |
| Brookings Global Metro Monitor | <https://www.brookings.edu/research/global-metro-monitor/> |
| Urban Age Data | <https://urbanage.lsecities.net/data> |
| GRUMP - Global Rural-Urban Mapping Project | <https://sedac.ciesin.columbia.edu/data/collection/grump-v1> |
| Metropolis Observatory | https://indicators.metropolis.org |
| World Council on City Data | [https://open.dataforcities.org](https://open.dataforcities.org/) |
| Word Pop | https://www.worldpop.org |
| World Urban Database and Access Portal Tools | www.wudapt.org |
| PWC Cities of Opportunities | www.pwc.nl/nl/assets/documents/pwc-cities-of-opportunity |
| Global Power City Index | www.mori-m-foundation.or.jp |
| The Economist's Global Liveability Index | https://www.eiu.com/topic/liveability |
| GaWC City Classification | https://www.lboro.ac.uk/gawc/world2018t.html |
| Global Cities Lab Top 500 | http://globalcitylab.com/ |
| JLL`s City Momentum Index | https://www.us.jll.com/en/trends-and-insights/research/city-momentum-index-2020 |
