# Supplementary figures and images for: An introduction to DUIA: The database on urban inequality and amenities
Source: PLoS One. 2021 Jun 25;16(6):e0253824. doi: 10.1371/journal.pone.0253824 (PMC8232421; doi:10.1371/journal.pone.0253824)

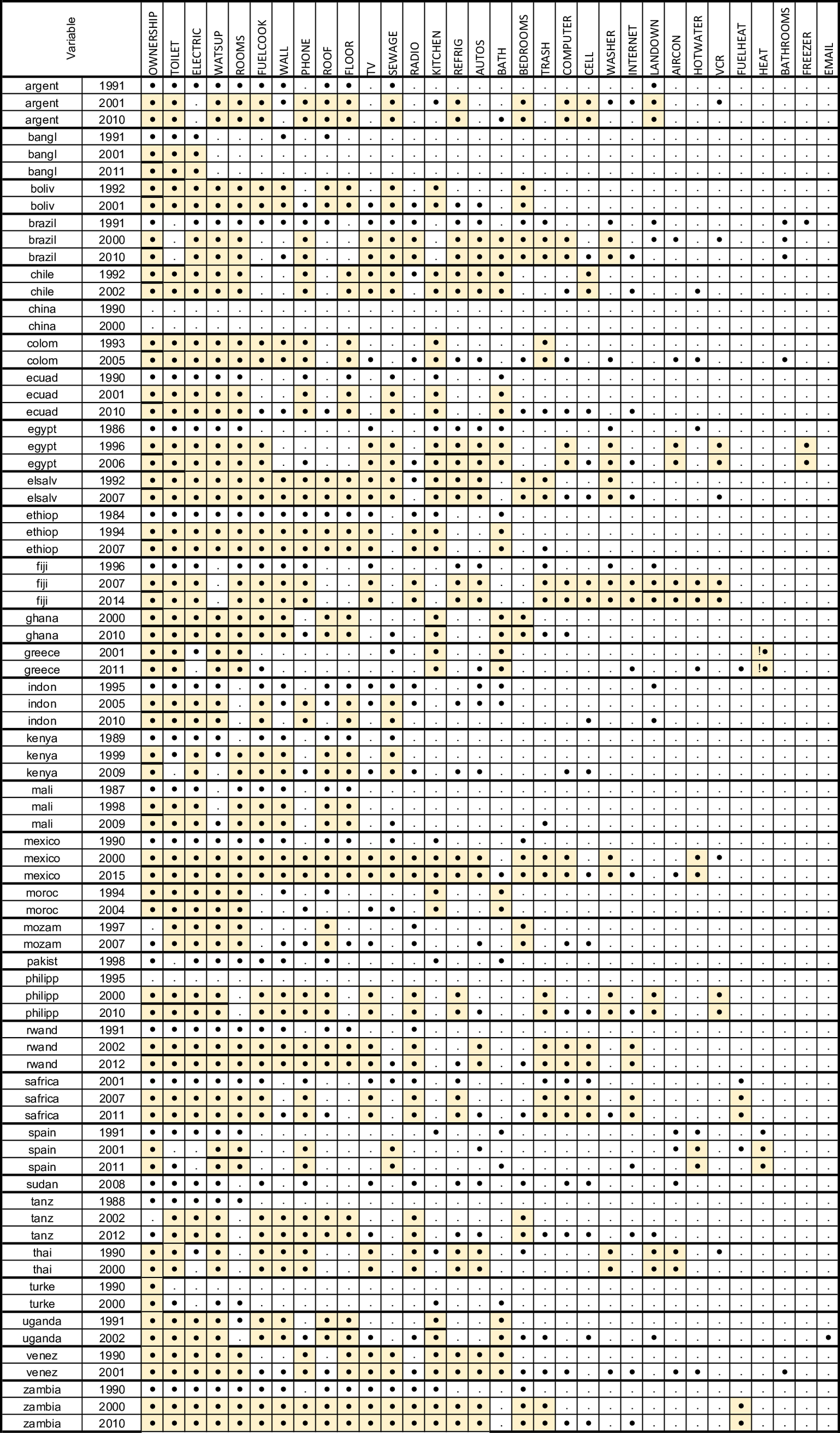

Supplement: S2 Appendix — (TIF) [file pone.0253824.s002.tif]
