# Supplementary material for: An introduction to DUIA: The database on urban inequality and amenities
Source: PLoS One. 2021 Jun 25;16(6):e0253824. doi: 10.1371/journal.pone.0253824 (PMC8232421; doi:10.1371/journal.pone.0253824)
Supplement: S3 Appendix — (DOCX) [file pone.0253824.s003.docx]

## S3 Appendix. Delineating cities: the examples of Mexico and Dhaka

Figure bellow visualizes the spatial match between the geographical layers of interest: the administrative unit as defined in the census and the urban extent as defined by the AUE. The administrative polygons layer is filled in light gray while the AUE study area limit is represented by the red line. Degrees of yellow indicate the urban footprint in different periods. On the right side of the figure, the example of México City illustrates the situation where AUE study area perfectly matches the administrative units. On the left side of the figure, the city of Dhaka represents an example of a mismatch. The decision to be made in cases like Dhaka is whether to include or exclude administrative units that are partly outside the city limits as defined in the AUE. We decided to include such units: we prefer to include households that do not reside in the city over excluding households who do reside in the city. Another difference is the number of second administrative units that subdivide each city. México is divided into 60 units while Dhaka is divided into only three. This means that the possibilities to perform intraurban analysis vary considerably.

**Spatial match IPUMS Enumeration Areas (Level 2) and AUE study area in Dhaka and Mexico City**


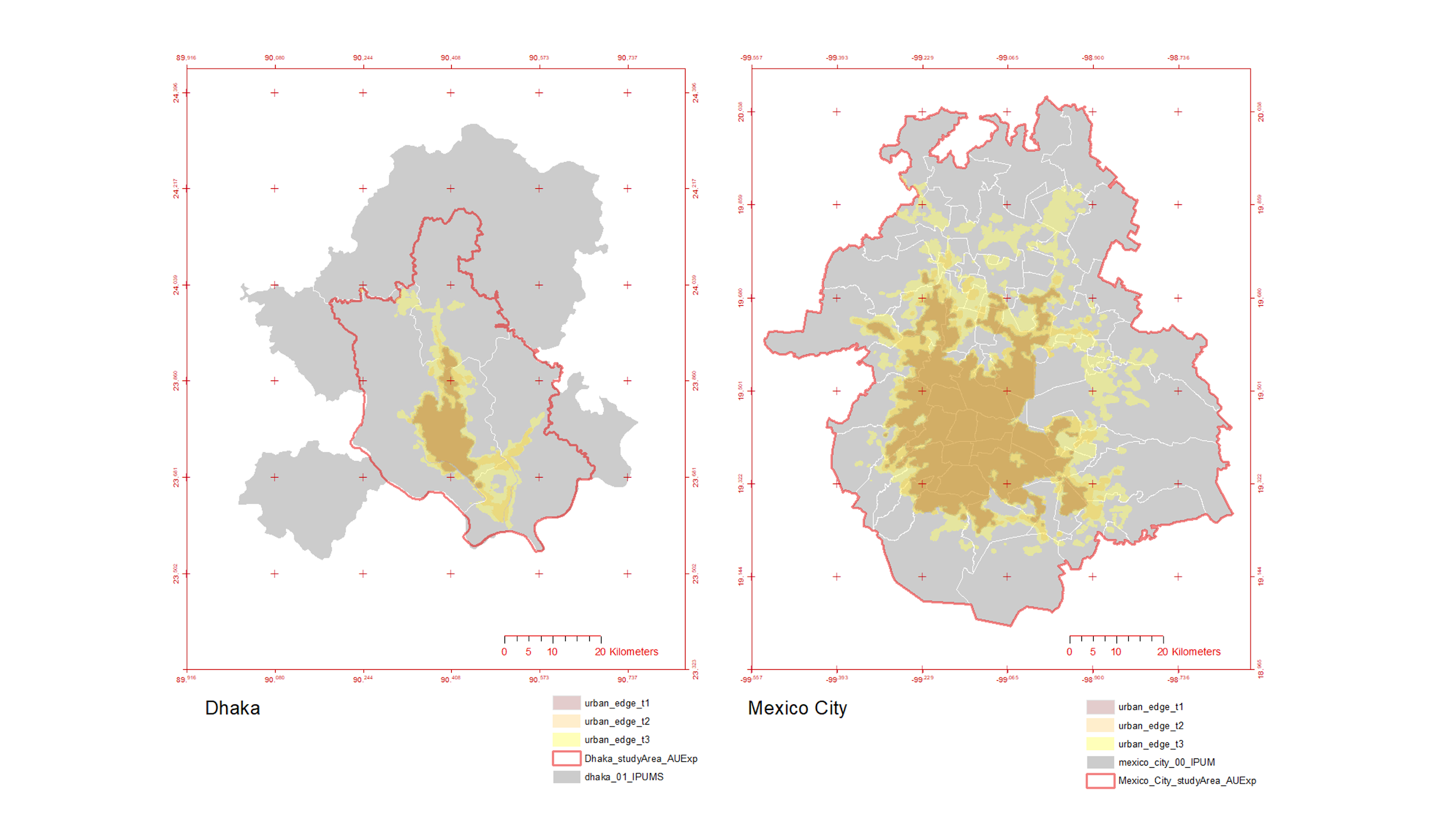


These differences in terms of scale and coverage are behind the Modifiable Areal Unit Problem (MAUP). Although the MAUP is inherent in any analysis based on aggregated data and therefore cannot be fully resolved, DUIA at least makes explicit which choices were made regarding spatial coverage, scale, and coupling between data sources. Moreover, it is possible to modify our choices by revising the R code.
