# Supplementary material for: An introduction to DUIA: The database on urban inequality and amenities
Source: PLoS One. 2021 Jun 25;16(6):e0253824. doi: 10.1371/journal.pone.0253824 (PMC8232421; doi:10.1371/journal.pone.0253824)
Supplement: Attachment — Submitted filename: DUIA General and Review Comment.docx [file pone.0253824.s004.docx]

**General Comment**

This work is a clear presentation of what is on the ground. But the database can be used to show how inequality will be in time to come for the selected cities. Therefore the analysis can be extended to include projections for the selected cities or he can make it a recommendation for further research.

**Review comment**

He should review the work and add the suggestions.
