# Supplementary material for: An introduction to DUIA: The database on urban inequality and amenities
Source: PLoS One. 2021 Jun 25;16(6):e0253824. doi: 10.1371/journal.pone.0253824 (PMC8232421; doi:10.1371/journal.pone.0253824)
Supplement: Attachment — Submitted filename: rebuttal letter PLOS ONE.docx [file pone.0253824.s005.docx]

Dr. Frederico Ramos

University of Amsterdam

Human Geography, Planning and

International Development - GPIO,

Nieuwe Achtergracht 127, 1018 WS Amsterdam, The Netherlands

May 23, 2021

Rebuttal letter

Dear Academic Editor and Reviewers,

We thank you for the comments on our manuscript titled *An introduction to* *DUIA: The database on urban inequality and amenities*. All comments were very good and are under consideration in our revision. Please, find below our point-by-point response to the observations and suggestions raised by the editor and reviewers. We hope that we addressed them satisfactorily.

**Academic editor:**

1. *Please ensure that your manuscript meets PLOS ONE's style requirements, including those for file naming.*

We have carefully checked the templates and made the adjustments to meet the journal requirements.

2. *We note that Figure 1 and S3 Appendix in your submission contain map images which may be copyrighted. All PLOS content is published under the Creative Commons Attribution License (CC BY 4.0), which means that the manuscript, images, and Supporting Information files will be freely available online, and any third party is permitted to access, download, copy, distribute, and use these materials in any way, even commercially, with proper attribution. For these reasons, we cannot publish previously copyrighted maps or satellite images created using proprietary data, such as Google software (Google Maps, Street View, and Earth).*

All figures were produced by the authors specifically for this manuscript and no copyrighted images were used in their production.

3. *Please include captions for your Supporting Information files at the end of your manuscript, and update any in-text citations to match accordingly.*

We have checked the templates and made the adjustments to meet the journal requirements.

**Reviewer #1:**

4. *Please address the following minor corrections.*

We have addressed all the indicated minor corrections accordingly.

**Reviewer #2:**

5. *My point is that the database can be used to show how inequality will be in time to come for the selected cities. Therefore, the analysis can be extended to include projections for the selected cities.*

We have included a paragraph in the section ‘Limitations and opportunities ahead’ to discuss possible applications, including econometric analysis using microdata level. Nevertheless, we are skeptical about the potential to conduct robust projections mainly due to the sparse time data availability.

**Reviewer #3:**

6. *In the introduction, the authors raised some important limitations of existing databases (lines 46-58). However, they failed to reference any specific databases or include any citations to support these claims. While some examples are discussed in later sections of the paper, a summary in the introduction will be useful to readers. I recommend that the authors should refer to specific databases as examples in discussing these limitations.*

We have included the references in the introductory section as suggested.

7. *Where is the database? The authors described how they extracted and integrated data from the two sources they included in deriving the database. However, they failed to describe the outcome of that process. A section that clearly explains the features of the database will be helpful. This section should be presented before the illustrations.*

We agree and incorporated a new subsection in the third section (before the illustrations) describing the components and features of the database. This new subsection highlights the use of a Github repository for the R codes that enables replication and the use of a Figshare webpage to distribute the aggregated-level database. We will provide and include the links and respective DOIs after passing the point of anonymous peer review.

8. *Similar to my comment above, the authors should discuss the application of the database in-depth. The two illustrations are good but are there other applications that you anticipate? I recommend that somewhere, either in the conclusion or limitations, the authors should clearly discuss the nature of uses they anticipate for their database.*

As responded to reviewer #2, we included a paragraph in the section ‘Limitations and opportunities ahead’ dedicated to possible applications. These applications include multivariate statistical analysis, logistical regressions, and comparative analysis.

Sincerely yours,

Dr. Frederico Roman Ramos (corresponding author) and Prof. Dr. Justus Uitermark

E-mail: f.ramosroman@uva.nl
